# Multimorbidity Development in Working People

**DOI:** 10.3390/ijerph16234749

**Published:** 2019-11-27

**Authors:** Sukyong Seo

**Affiliations:** College of Nursing, Eulji University, Seongnam 13135, Korea; sue.seo@gmail.com; Tel.: +82-31-740-7128

**Keywords:** multimorbidity, health disparities, working population, prevalence, chronic disease, Korea Health Panel, SES

## Abstract

Multimorbidity is defined as the coexistence of multiple chronic conditions in one person. It affects the way people lead their lives and might be a heavy burden, especially for those with limited material resources. This study explores the prevalence of multimorbidity in the working population and discusses the distribution of multimorbidity in specific sub-groups. We conducted a longitudinal analysis of nationally representative data in South Korea (Korea Health Panel, 2010–2015). Generalized estimation models were applied to examine the individual effect of socioeconomic status (SES) and job-related variables. We found that about five percent of workers who initially had no or one chronic condition developed multimorbidity during within five years. About 20% of working women had multimorbidity at age 55, about 10 years earlier than working men. A higher prevalence appeared in working women with school-age children, non-standard employment, no autonomy at work, or unskilled occupation. SES was significantly associated with a higher prevalence of multimorbidity in both gender after controlling for the effect of age and other covariates. Multimorbidity is a major health concern in the working population and prevention and control should be promoted in the workplace.

## 1. Introduction

Multimorbidity is defined as the presence of multiple long-term medical conditions within an individual. People with lower material resources are more likely to experience a greater burden in managing multimorbidity [1,2,3]. The working population can typically count on reduced personal and community resources (both economic resources and time availability); thus, they experience greater difficulty in effectively managing health problems. Managing a chronic disease, fatigue and activity limitations at work are perceived as stressful for workers with chronic diseases [4]. In particular, working with uncertainty and unpredictability of painful symptoms (e.g., arthritis) can be more stressful, or they often have greater perceived stress associated with future uncertainty, balancing out multiple roles, and difficulties psychologically accepting the impact of disease [5,6].

Research based on general population samples shows that multimorbidity is associated with higher mortality, poorer functional status, and quality of life [1,2,3,7]. People with multimorbidity use ambulatory and emergency care more often than those without multimorbidity [7], and they represent an increased burden on health care systems [1]. Recently, an increasing number of researchers have reported the impact of multimorbidity on occupational outcomes: workrelated stress [6], work-loss [4,8], or giving up labor market participation [9]. For example, Smith et al. explored Canadian workers and found that multimorbidity was significantly associated with increased probability of not working due to health reasons [9].

The prevalence of multimorbidity ranged from 3.0% to 30.0% [3]. Tezlaff and colleagues [10], for example, reported the prevalence rate was about 3.0 among the the working German population. The rate was lower than that obtained in a Canadian sample aged 45–49 (about 30.0%) [11]. Among Swedish, about 12% had two or more chronic conditions [12]. Related evidence on multimorbidity in the workforce is scarce in East Asian countries.

Multimorbidity is strongly associated with low socioeconomic status (SES) [1,2,3,4,5]. For example, Katikireddi et al. reported that the risk of developing multimorbidity was approximately 1.5 times higher in poor than in rich individuals [2]. Research based on working population samples also reported a different prevalence of multimorbidity across SES [3,13,14]. The workers’ health outcome was better when they had autonomy over occupational tasks and schedules [14], or they had a highly qualified job [13].

In addition, women have been found to have a higher risk of multimorbidity than mem in many studies [1,2,3,11]. This gender difference may be responsible for women’s greater longevity [3,10] or lower SES [11]. Little is known concerning whether the difference existed after controlling for the effect of age and SES. Few have investigated whether job characteristics or child-raising burdens may mediate health outcomes among women. Female workers with chronic conditions need to manage tasks associated with symptom prevention, diagnosis, and management, as well as those related to housework, childcare, earning money, and personal care. There is evidence that the work–life burden of female workers is related to health outcomes, including musculoskeletal disease, headache, and fatigue [1,10,11,13].

In sum, although multimorbidity has been examined extensively in general population samples and Western countries, relatively few studies have focused on the working population, particularly in East Asian countries. The objectives of this study were: (1) to address the comprehensive assessment of multimorbidity among working people in Korea; (2) to examine if SES and job-related variables were significantly associated with the development of multimorbidity; and (3) to contribute to filling the gap in the literature by paying attention to gender inequalities in multimorbidity. We hypothesized a higher rate of multimorbidity prevalence appeared and the health inequality was considerable across workers because of employment conditions in Korea. The Korean economy has been under severe recession due to the global financial crisis 2007–2008, which was accompanied by a severe blow to the labor market: non-standardized employment arrangement has been prevalent and the economic inequality between workers growing. Nearly 80% of Korean workers reportedly believe that the economic crisis has had a detrimental effect on their health [15]. Working women’s health outcomes may be worse in Korea with Confucian traditions regarding the roles of women, which emphasize filial piety, seniority, and the responsibility of married women to their parents-in-law. They may experience greater struggles to manage their health issues and life responsibilities at work and home than women in Western countries. The worry that gender difference is widening has been voiced [16].

## 2. Methods

### 2.1. Data

Our study was a longitudinal analysis of a population-representative dataset extracted from the Korea Health Panel (KHP) survey (2010–2015) [17]. The survey started in 2008 and has been repeated annually since then. Based on the Population and Housing Census in 2005, the initial KHP sample was designed as a nationally representative cohort of non-institutionalized men and women living in South Korea across all age groups. Attrition since the first wave has occurred among specific groups, including non-homeowners, less affluent households, younger people, and highly mobile individuals. Thus, in our analysis, we applied pot-stratification weights calculated to adjust for these attritions [17].

**Figure A1:**
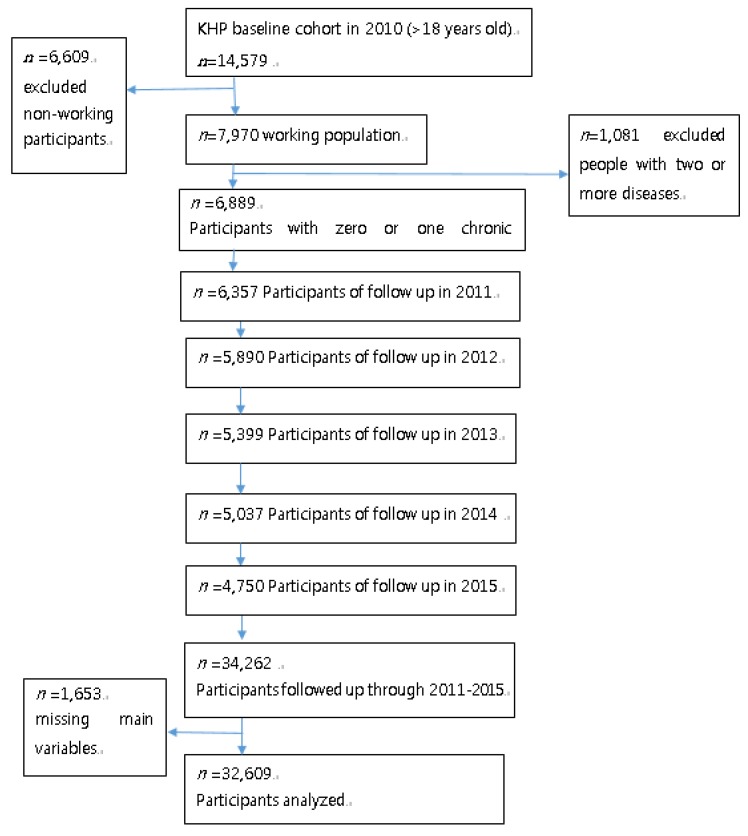
Flow-chart of the study population.

An unbalanced panel of 6889 individuals with no chronic disease or one chronic condition (at the baseline year of 2010) was constructed after excluding people aged below 18 years and followed up for five years, including our analytic data with 34,262 person-year observations. Participants were followed for an average of 3.6 years (range: minimum of 1 year to a maximum of 5 years). All the 2139 drop-outs occurred through 2011–2015. Our analytic cohort had year-on-year loss rates of about 5.2% (7.7% in 2011; 8.3% in 2012; 7.4% in 2013; 6.7% in 2014; 5.7% in 2015). The original cohort had a drop-out rate of approximately 5.0% [17]. A flow-chart of the study population is presented in Appendix A Figure A1.

The survey data were collected through face-to-face interviews using self-report questionnaires regarding demographic information, health behavior, and current health status. The interviewer asked whether the survey participants had a chronic disease, whether they were diagnosed by a doctor, whether they had any medical document for long-term conditions, and whether they were taking medication for chronic conditions. ICD-10 codes for chronic diseases were recorded by the interviewer based on the information. The data are available from Korea Institute for Health and Social Affairs (KIHASA) under special permission for research purposes. Ethical approval for this data was obtained by KIHASA.

### 2.2. Variables and Measurement

The dependent variable of the present study, multimorbidity, was defined as the presence of two or more chronic conditions in one individual. There is no standard method for measuring multimorbidity [3]. In order to capture a comprehensive picture of disease pattern, we selected the most frequent chronic conditions with a prevalence ≥ 1% among the working population found in the KHP data set during the study period (Appendix B Table A1), following Bussche et al. (2011) [18]. Obesity was on Bussche and colleagues’ list, but it was not recorded as a disease in Korea. Calculating the body mass index (BMI) for all participants using the data on using self-reported weight and height, we defined obesity as being >/= 30 kg/m^2^ and classified as one chronic condition.

Job-related variables included three measures: (1) standard employment-based versus a non-standard employment-based job, (2) autonomy versus non-autonomy at work, and (3) occupation type. Non-standard workers represent temporary employees and daily workers and often work under poor employment conditions such as low wages, limited fringe benefits, and deficient job security. They take up more than half of paid workers in Korea. The variable of occupation type was defined based on an occupation classification system developed by Blossfeld [19]. In the present study, following Tezlaff et al. [10], the original 12 groups were re-categorized into four groups: highly qualified, specialist, skilled, and unskilled.

We included income and education to measure socioeconomic status. The KHP survey collects information on total household income from employment, pensions, investment, and savings for all house members. Our income variable was adjusted by dividing total household income by the square root of the number of household members during the current year. We obtained three groups, and individuals were ranked from 1 (the lowest income group) to 3 (the highest income group) based on the adjusted household income variable. Our data provided a variety of income measures including current income level, home ownership, and home values. We included the current income level in our final model, as the rest did not have significant bivariate associations with multimorbidity development. The Wald F-statistic for income level was statistically significant (*p* < 0.05).

People were also classified into three groups based on their educational level: elementary school graduates or lower, high school graduates or lower, and college or above. The categorical variable of education was converted using the continuous variable (the number of years) provided in the original data set and both showed similar results.

This study included gender and family structure to measure gender inequalities. Family structure was assessed according to whether the participants were married and had school-age children. Further, participants with school-age children were asked if they experienced stress related to educating their children. The variable of child-raising burden was measured using their answers to this question.

Control variables, which would be related to explanatory and outcome variables, were selected by referring to previous studies and included age, health behavior, and unmet health care need. The health behaviors considered as factors in this study included: smoking habits, alcohol consumption, and physical activity. The data on these factors provided by the participants, using standardized self-report questionnaires. Participants were classified as current smokers or non-smokers. Their consumption of alcohol was determined based on the intake frequency over the past year, at least once a month or never. Participants were questioned on their leisure time and work-related physical activities. Their responses were classified either as practicing moderate physical activity at least once a week or never. The variable of unmet health care needs was measured by the respondents being asked whether, in the past 12 months, they ever felt that they needed health care services, but they could not receive them. We assessed for a bivariate relationship between each of the possible covariate variables and our dependent variable before including it in the final model. For example, in theory, having a usual source of care determines multimorbidity development [3], but no significant association was found in our data. Additionally, upon testing regional dummies and private insurance enrollment as proxies of access to care, we did not find any significant results. We, therefore, decided to include unmet health care needs in the final model.

### 2.3. Statistical Analysis

We described baseline characteristics using means and proportions and conducted the Chi-square test to analyze baseline differences between men and women. The prevalence of multimorbidity in the working population was analyzed across gender, SES, and occupation groups. First, we estimated crude rates of multimorbidity prevalence through 2011–2015 and looked at the prevalence trend over time. Two statistical methods were applied: Poisson regression and a set of linear generalized estimation equation (GEE) models. Since this study examined whether people who had zero or one disease at baseline developed multimorbidity in the consecutive years, the prevalence estimates did not represent the prevalence of multimorbidity among the general working population (those free of the disease should be the denominator for calculating the prevalence). The prevalence rate was calculated using a Poisson regression (number of event/persons-time) using a stata command “sptime”. To identify the factors associated with multimorbidity development, we performed a multivariate logistic regression analysis accounting for repeated observations of the same individuals over time. For dichotomous dependent variables, logistic GEE models are appropriate. Separate analyses were also conducted for working women and working men. Statistical analyses and data management were performed using STATA 13.1 (StataCorp, College Station, TX, USA).

## 3. Results

### 3.1. Sample Description

**Figure 1:**
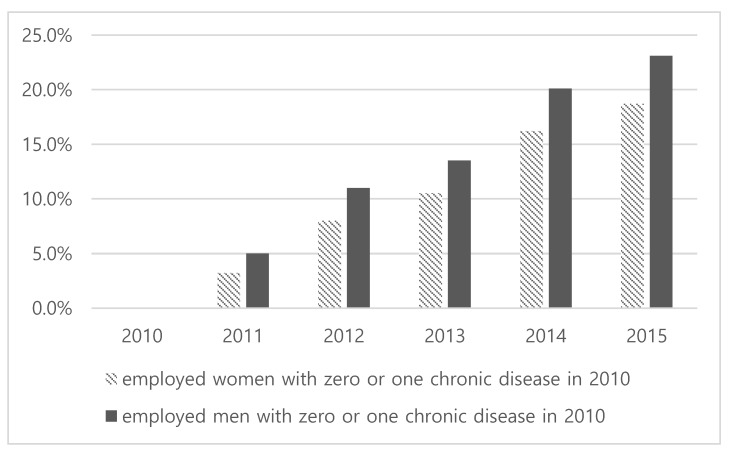
Trend of multimorbidity prevalence in working population with zero or one chronic disease in 2010 (Korea Health Panel, 2010–2015).

**Figure A2:**
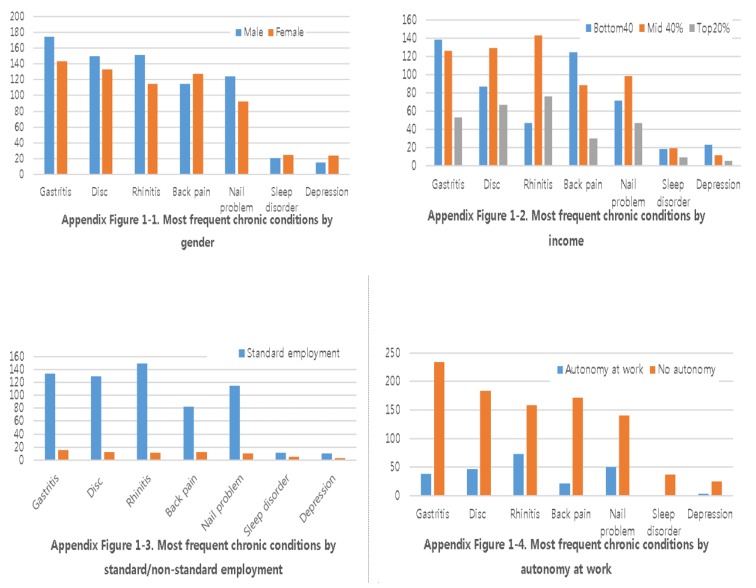

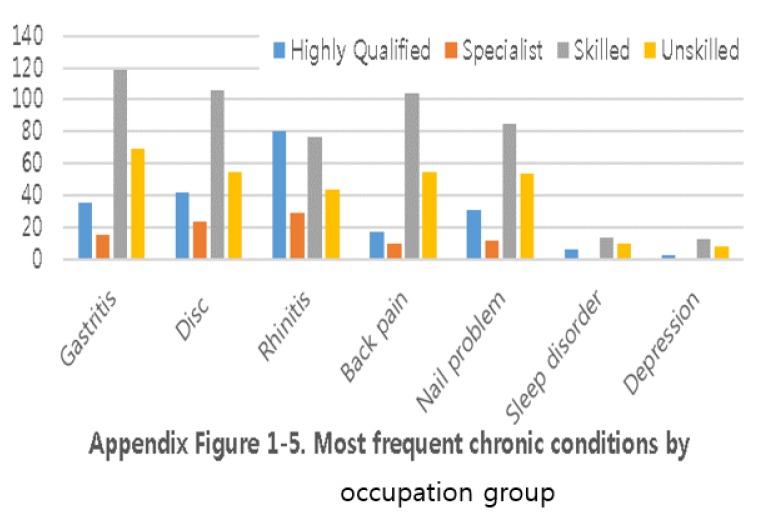
Most frequent chronic conditions in Korean workers developing multimorbidity in 5-year follow-up.

The characteristics of the study sample are presented in Table 1. A total of 6889 participants contributed to 32,609 person-year observations of follow-up between 2010 and 2015. Women (39.8%) were considerably fewer than men (60.2%) in our sample. The mean age was 45 years old. Those with standard employment-based jobs were the most common. More than half of the workers reported they had no autonomy at work. The skilled occupation variable had twice as many men as women. A proportion of respondents belonged to the bottom 40% was higher in men (59.2%), whereas educational levels tended to be higher for men. About half of the workers had school-age children. With regard to the variable concerning unmet health care needs, the percentage of women (37.8%) was lower than men (62.2%). A substantial number of working women reported physical inactivity (70%). The proportion of individuals with multimorbidity increased over time for both women and men (see Figure 1). Since we studied people who have zero or one disease at baseline and then investigated whether they developed or not multimorbidity in the consecutive years, the measured prevalence did not represent the prevalence of multimorbidity in the general working population free of morbidity (who should be the denominator for calculating the prevalence). The distribution of diseases found in the population and in specific sub-groups was presented in Appendix B Figure A2 and Table A1.

### 3.2. Prevalence of Multimorbidity

**Figure 2:**
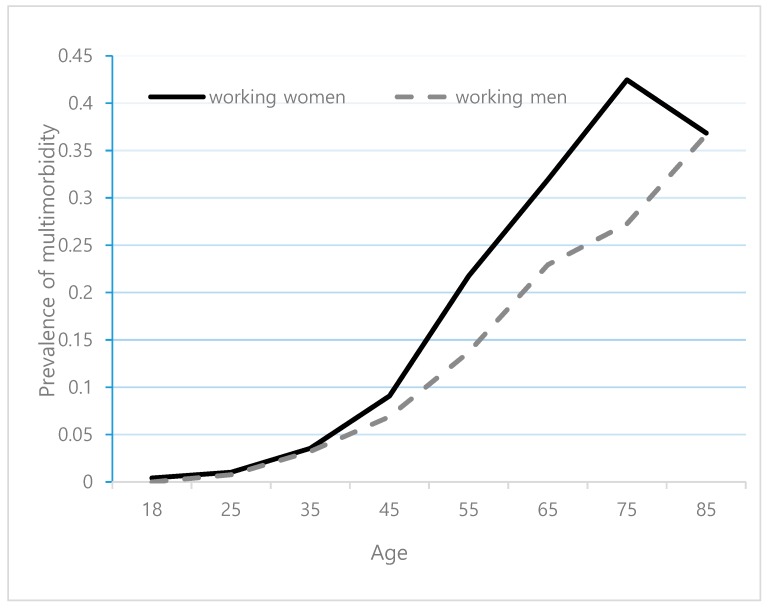
Prevalence of multimorbidity by age in working population using the Korea Health Panel (2010–2015).

The prevalence of multimorbidity increased steadily with age, and it was higher in women than men (Figure 2). About 20% of working women had multimorbidity at age 55, which was about 10 years earlier than men.

Table 2 shows the prevalence rates per 100 person-years of multimorbidity by group. We found 4.88 (95% CI: 4.61–5.16) recorded as the overall prevalence. The prevalence rate for participants over the age of 55 was 10.59 (95% CI: 9.83–11.42). It was higher among elderly women (13.44 among female and 8.96 among male elderly). We found an inverse relationship between multimorbidity development and income. Educational levels also had an inverse relationship. These socioeconomic differences existed for both women and men. Having less education increased multimorbidity risks by 126% in men. The prevalence rate was higher among people with non-standard employment, with no autonomy at work, and unskilled workers. The difference across occupation groups was prevalent in women.

### 3.3. Regression Results

The GEE logistic models using two or more chronic conditions versus no or one condition as outcome variable showed that multimorbidity prevalence rates increased over time (see Table 3). Age was significantly associated with multimorbidity, but job-related variables showed no significant association. These results held for working women and men. High income showed no significant difference compared to low income in men. A significant association with educational level was observed. In both genders, a clear effect of educational level on multimorbidity risks was observed. Marital status and child-caring burden were not significantly associated with multimorbidity in women. 

## 4. Discussion

Using longitudinal data, we were able to examine how multimorbidity developed within the working population. This enabled us to apply a panel data analysis for controlling for the effect of time-invariant individual characteristics on the development of chronic diseases. Further, the sample was representative of the general population, and an occupation variable was collected in a standardized manner for each year. We focused on the inequalities related to gender, SES, and job-related factors, which has not been adequately addressed in previous studies [11]. As far as we know, this study is the first to examine the impact of various occupational factors on multimorbidity development in East Asian countries.

This study found an increasing trend of multimorbidity development in the working population using national representative data from South Korea. This is in line with previous studies [10,11]. Our data revealed a prevalence rate of 4.88 per 100 person-years with a mean age of 45 years. To compare these results with the data obtained by Dhalwani and colleagues [20] on an older English population with a median age of 61 (in which the prevalence rate was about 6.0), we restricted the data to the respondents aged 55 or older (the median age was 60 years, with an interquartile range of 57–65 years) and found a prevalence rate of 10.59. The measurement of multimorbidity used in Dhalwani et al. was similar to that of this study (≥2 diseases from 20 types of diseases). This rate was lower than that obtained in a cross-sectional study of a Malaysian sample (of about 13.0) performed by Hussin and colleagues [21], as well as of a Swedish cohort analyzed by Melis and colleagues [12] (of about 12.0). However, Melis et al. used different multimorbidity measures from this study (the number of conditions analyzed was 40 in Melis et al. and 23 in this study). Since prevalence estimates increase as the number of conditions included in the count increase, this study’s relatively lower prevalence rate could contribute to the differences in multimorbidity measurement methods.

Previous studies have produced evidence on the societal inequalities in multimorbidity development [1,2,7,10]. Our findings were also consistent with these findings. The prevalence rate was higher among women (5.55 per 100 person-year) than men (4.44 per 100 person-year), by about 25%. Workers in the bottom 40% income group had a higher prevalence rate (6.91) than that of the top 20% income group (3.91). Consistent with a stepwise gradient between income and multimorbidity presented in previous studies, there was a significant linear relationship among women (albeit not among men) in our GEE models [1,2,3,10]. The educational level also had a clear inverse relationship after controlling for the effect of income and covariates.

In our analysis, the associations between multimorbidity and income were not significant in men less consistent than with educational level. This could indicate that the differences among male workers were better represented by educational level than income. Both income and educational level are well-established indicators of SES, but may not equally reflect relevant aspects of social inequality [22,23]. In the working population, educational level could reflect life-long social inequalities that go beyond income [24]. In this study, income is only represented by current earnings, which does not seem to be an appropriate proxy for long-term income, or permanent wealth, and persistent or transient poverty [23]. The differential impact of educational level and income might indicate that the education variable measured a specific effect of different living conditions in the working population with multimorbidity, which is more closely related to multimorbidity.

Differences were also found for occupational subgroups. The prevalence of multimorbidity increased in workers with non-standard employment, with no autonomy at work, and in people with higher qualification and skilled occupation. However, in the GEE analysis, multimorbidity risks did not decrease with any of those job-related variables. The difference could contribute to the relatively small size of our cohort as well as a short period of duration, which hindered wider variations for time-varying job-related factors used in regression models. Future studies need to employ another data source with a longer cohort.

Previous studies also showed controversial findings. In Tezlaff et al. using German data, workers with higher qualification had a lower risk of developing multimorbidity compared to unskilled workers [10]. According to a recent study on South Asian adults, however, highly qualified service workers had a higher risk of suffering from multimorbidity compared to manual laborers. The authors reported that the higher risk in qualified workers could potentially be due to occupation-related physical inactivity, sitting time, dietary factors, or a variety of other health effects found among unskilled workers. Future studies need to control for potential health effects found among workers to examine whether job-related variables have a clear impact on multimorbidity development.

Including child-raising burden and age in the same models may have introduced error into the models, as younger people are more likely to have school-aged children, so these two variables can be highly correlated. We performed regression analysis including the interaction terms of child-raising burden and age group. The interaction should show a relative risk between people with children and those without children. We found significantly higher ORs (2.64, 95%CI: 1.39–4.98) in the younger female working population (<45 years old), but no significance among middle-aged female workers (45–55 years old). A similar result appeared for men. These findings point to a significant impact of child-raising burden on multimorbidity risk.

We should note several limitations of this study. We only included data from the six most recent KHP waves for this analysis. Although we still had a large number of people with about 30,000 person-years of follow-up, the number of people with some specific conditions of covariates such as smoking and binge drinking was quite low, depicted by wide confidence intervals of the estimates. Further, as mentioned above in the Method section, our cohort data (2010–2015) tended to have some attrition since the first wave among non-homeowners, less affluent households, younger people, and highly mobile individuals. This may have affected our findings. First, non-homeowners or less affluent people in the working population may be at a high risk of developing co-morbidities. Given the relationship between poverty and health outcomes, our sample would consist of individuals at a relatively higher risk. Second, young people may be at a low risk for chronic diseases. It is, therefore, possible that our findings were underestimated or overestimated. Accordingly, the analysis has limited statistical power, and the findings should be interpreted with caution.

Our regression analysis included a set of variables of health behavior (smoking, alcohol drinking, and physical inactivity) as a control variable and showed no significant coefficient for most variables of health behaviors except physical inactivity, contrary as expected. This result, however, does not imply that the behavioral risk factors have no causal relation with multimorbidity in the working population. There may exist a health selection effect when we limit the analysis to the working population. Lifestyle factors could explain the disparities within gender groups and occupation classification found in our analysis [25]. Future studies need to examine whether the effect of health behaviors could widen the estimates on disparities in the prevalence rates of multimorbidity.

Defining multimorbidity as the presence of at least 2 chronic conditions in the same person, we used the list of the 23 most frequent chronic conditions with a prevalence ≥1% in this study sample. As mentioned earlier, this measure is similar to that of previous studies [18,20]. However, there is no consensus on how to measure multimorbidity. In particular, the number of conditions counted differed in previous studies. According to a systematic review, the number of conditions analyzed ranged from 5 to 335 [3]. Unsurprisingly, the measured prevalence of multimorbidity increased as the number of conditions included increased (12.9% to 95.1%). As a result, the comparison of prevalence across studies using different methods for measuring multimorbidity presence should be done carefully. One way to solve the problem is to examine changes in the prevalence of multimorbidity over time, using the same measurement method in a consistent population sample [10,20].

Finally, our analysis did not examine whether the result changed for a subgroup by the type of disease. Women are more likely to have depression [26], which could be observed in working women with low SES [1]. If we limit the analysis to participants with any mental health disorders, excluding people with physical diseases, our findings on the gender disparities may or may not change. This lack of specificity may hinder the statistical analysis and detract from the overall public health impact of the findings. Future studies need to investigate whether working people are more vulnerable, depending on the type of multimorbidity that they experience, and provide critical information about which demographic groups would need targeted interventions or treatments for specific diseases.

## 5. Conclusions and Policy Implications

We found that multimorbidity was a common phenomenon in the working population, and women experienced a greater burden of multimorbidity. These findings illustrate the importance of multimorbidity as a major public health concern. Patients with multimorbidity often have a combination of physical, psychological, and social problems, and they need time, empathy, and a holistic patient-centered approach to care [27]. However, in South Korea, many essential services are not covered by national health insurance, and coordination of primary care is very limited [28]. Very weak incentives are provided for health care providers when improving the health records of patients. Since these providers are compensated on a fee-for-service (FFS) basis, they are likely to increase their numbers of patients and reduce time spent on counseling and teaching self-management to their patients. Individuals with low SES and multimorbidity in South Korea are likely to experience unmet needs. Thus, policymakers should prioritize chronic disease management for people with low SES.

The increase in the prevalence of multimorbidity is also a challenge for health care providers, who are typically trained based on a single disease-oriented medical curriculum. Policies directed at identifying appropriate health professionals and preparing them to meet the complicated needs of patients are necessary to effectively deal with the increasing prevalence of multimorbidity.

Finally, we emphasize the importance of promoting the prevention and control of chronic diseases in the workplace. Recent governmental efforts to build a national chronic disease management system have been based on a public health center or a community service center and operated daytime programs. We recommend focusing more on providing services in the workplace, which can be more easily accessed by the working population.

## Figures and Tables

**Table 1:** Baseline characteristics of the study population from the Korea Health Panel (2010).

Variables *n* (%) Mean (SD)	Total	Female	Male	*t, Chi* ^2^
	*n* = 6889 (100.0)	*n* = 2743 (39.8)	*n* = 4146 (60.2)	
Age (years)	45.4 (12.49)	44.1 (0.25)	46.3 (0.19)	−7.20 ***
Age group				
<45	3513	1487 (42.3)	2023 (57.7)	19.32 ***
45–55	1934	729 (37.7)	1205 (62.3)	
>55	1442	527 (36.5)	915 (63.5)	
Standard employment-based job				
Standard	4418	1735 (39.3)	2683 (60.7)	10.33 ***
Non-standard	373	178 (47.7)	195 (52.3)	
Others	2098	830 (39.6)	1268 (60.4)	
Autonomy at work				
Autonomy	1418	230 (16.2)	1188 (83.8)	523.36 ***
No autonomy	4260	1928 (45.3)	2332 (54.7)	
Do not know	550	357 (64.9)	193 (35.1)	
Occupation type				
Highly qualified	1650	613 (37.2)	1037 (62.8)	228.98 ***
Specialist	812	428 (52.7)	384 (47.3)	
Skilled	2731	847 (31.0)	1884 (69.0)	
Unskilled	1696	855 (50.4)	841 (49.6)	
Income				
Bottom 40%	1704	695 (40.8)	1009 (59.2)	4.32
Mid 40%	3320	1280 (38.5)	2040 (61.5)	
Top 20%	1865	768 (41.2)	1097 (58.8)	
Education				
Elementary	1610	797 (49.5)	813 (50.5)	90.29 ***
High school	2530	983 (38.8)	1547 (61.2)	
College+	2749	963 (35.0)	1786 (65.0)	
Marital status				
Married	5274	1897 (36.0)	3377 (64.0)	139.03 ***
Unmarried	1615	846 (52.4)	769 (47.6)	
Having school-age children				
Yes	3574	1377 (38.5)	2197 (61.5)	10.89 ***
No	3142	1335 (42.5)	1807 (57.5)	
Unmet health care needs				
No	2552	1148 (45.0)	1404 (55.0)	44.42 ***
Yes	3086	1168 (37.8)	1918 (62.2)	
No need	965	333 (34.5)	632 (65.5)	
Currently smoking				
No smoking	4671	2650 (56.7)	2021 (43.3)	170.0 ***
Smoking	2045	62(3.0)	1983(97.0)	
Binge drinking				
Never	4212	2316 (55.0)	1896 (45.0)	100.0 ***
Sometimes+	2504	396 (15.8)	2108 (84.2)	
Physical activity				
No	2526	860 (34.0)	1666 (66.0)	67.50 ***
Sometimes+	4190	1852 (44.2	2338 (55.8)	

*** *p* < 0.01.

**Table 2:** Prevalence of multimorbidity per 100 person-time among the working population (2011–2015).

Variables		Prevalence Rate Per 100 Person Year (95% CI)					
		Total		Female Workers		Male Workers	
Overall		4.88	(4.61–5.16)	5.55	(5.10–6.03)	4.44	(4.12–4.79)
Age group (years)	<45	1.60	(1.38–1.86)	1.35	(1.05–1.73)	1.79	(1.48–2.16)
	45–55	4.64	(4.19–5.14)	5.72	(4.92–6.64)	3.98	(3.46–4.58)
	55+	10.59	(9.83–11.42)	13.44	(12.03–15.00)	8.96	(8.09–9.93)
Income	Bottom 40%	6.91	(6.28–7.61)	8.46	(7.37–9.70)	5.87	(5.13–6.72)
	Mid 40%	4.42	(4.06–4.81)	5.00	(4.40–5.69)	4.06	(3.63–4.54)
	Top 20%	3.91	(3.47–4.41)	3.94	(3.27–4.75)	3.89	(3.33–4.55)
Education	Elementary	9.87	(9.05–10.76)	11.31	(10.08–12.70)	8.48	(7.44–9.66)
	High school	4.80	(4.38–5.25)	5.30	(4.62–6.08)	4.48	(3.97–5.05)
	College+	2.38	(2.10–2.71)	1.63	(1.26–2.11)	2.78	(2.41–3.22)
Marital status	Married	5.36	(5.05–5.70)	5.97	(5.43–6.57)	5.02	(4.64–5.43)
	Unmarried	3.18	(2.74–3.68)	4.47	(3.74–5.32)	1.90	(1.45–2.49)
Having schoolage children	Yes	3.93	(3.61–4.28)	7.07	(6.35–7.88)	3.76	(3.36–4.20)
	No	6.18	(5.74–6.67)	4.22	(6.69–4.82)	5.54	(4.99–6.15)
Standard employment	Yes	3.65	(3.34–3.98)	4.32	(3.78–4.94)	3.26	(2.90–3.67)
	No	5.79	(4.57–7.35)	7.02	(5.02–9.83)	4.93	(3.52–6.90)
Autonomy at work	Yes	3.38	(2.93–3.91)	2.83	(1.91–4.19)	3.49	(2.98–4.07)
	No	5.17	(4.83–5.52)	5.80	(5.27–6.38)	4.67	(4.25–5.13)
Occupation type	Highly qualified	2.83	(2.42–3.32)	2.26	(1.67–3.05)	3.15	(2.61–3.80)
	Specialist	2.46	(1.91–3.16)	1.96	(1.30–2.95)	2.91	(2.12–4.00)
	Skilled	5.57	(5.11–6.08)	7.67	(6.66–8.82)	4.77	(4.27–5.32)
	Unskilled	5.90	(5.27–6.60)	7.09	(6.12–8.22-)	4.78	(4.02–5.69)

CI: Confidence Interval.

**Table 3:** Logistic generalized estimation equation (GEE)-regression on multimorbidity incident (two or more chronic diseases) in the working population (Korea Health Panel, 2010–2015).

Variables	Female Workers		Male Workers	
	Odds Ratio	95% CI	Odds Ratio	95% CI
Age group (referenced to 55+)				
<45	0.17 ***	(0.13–0.22)	0.30 ***	(0.24–0.37)
45–55	0.42 ***	(0.34–0.51)	0.58 ***	(0.49–0.68)
Standard employment-based job	0.79 *	(0.61–1.02)	1.08	(0.86–1.38)
Non-autonomy at work	1.05	(0.79–1.40)	0.95	(0.82–1.10)
Occupation type (ref. to unskilled)				
Highly qualified	1.15	(0.85–1.55)	0.99	(0.78–1.26)
Specialist	0.79	(0.55–1.15)	1.17	(0.85–1.60)
Skilled	0.93	(0.78–1.11)	1.04	(0.87–1.23)
Income (ref to. Bottom 40%):				
Mid 40%	0.84 **	(0.74–0.97)	0.96	(0.84–1.09)
Top 20%	0.84 *	(0.70–1.01)	0.88	(0.74–1.04)
Education (ref. to college+):				
Elementary school	2.95 ***	(2.12–4.12)	1.69 ***	(1.35–2.12)
High school	2.53 ***	(1.88–3.43)	1.48 ***	(1.26–2.38)
Married	0.96	(0.79–1.16)	1.85 ***	(1.43–4.11)
Having school age children	0.97	(0.79–1.19)	0.85 ***	(0.72–1.00)
Unmet health care needs (ref.to non-unmet need)				
Unmet needs	1.20 ***	(1.06–1.40)	0.95	(0.83–1.09)
No health care needs	0.51 ***	(0.30–0.84)	0.83	(0.62–1.09)
Currently smoking	0.92	(0.58–1.44)	0.74 ***	(0.65–0.85)
Binge drinking	0.83	(0.66–1.05)	0.90 *	(0.79–1.01)
Physical inactivity Year (ref.2011)	0.88 **	(0.79–0.99)	0.92	(0.82–1.02)
2012	2.31 ***	(1.93–2.77)	2.66 ***	(2.22–3.19)
2013	2.80 ***	(2.34–3.31)	3.39 ***	(2.83–4.05)
2014	4.65 ***	(3.88–5.57)	5.62 ***	(4.71–6.70)
2015	5.15 ***	(4.30–6.17)	6.40 ***	(5.36–7.65)
*N*	13,299		19,310	
Persons	2509		3683	
GEE correlation option	Exchangeable			
GEE family option	Binomial			
Wald F test	1065.24 ***		675.02 ***	

* *p* < 0.10, ** *p* < 0.05, *** *p* < 0.01.

## Data Availability

The datasets used and analyzed during the current study are available from the Korea Health Panel (KHP) survey by the Korea Institute for Health and Social Affairs [10] and the Korea National Health Insurance Service.

## Abbreviations

SES	Socioeconomic status
KHP	Korea Health Panel
KIHASA	Korea Institute for Health and Social Affairs
BMI	Body mass index
OR	Odds ratio
CI	Confidence interval
FFS	Fee-for-services

## Appendix B

Our stated aim was to investigate the distribution of multimorbidity and the inequalities of multimorbidity development by group in the working population of South Korea. It is also informative to discuss the distribution of diseases found in the population and in specific sub-groups (e.g., women, low income, less autonomy at work). To provide critical information about which demographic groups would need targeted interventions or treatment for specific types of diseases, we presented in Appendix the most frequent chronic conditions in Korean workers in five-year follow-up. Hypertension, diabetes, arthritis, and hyperlipidemia were the most prevalent in every subgroup. The rest of the chronic diseases were presented below. Men were more likely than women to have gastritis, disc hernia, or chronic rhinitis, whereas less back pain, sleep disorder, or depression. hypertension and/or heart disease. The low-income group was more likely to have gastritis or back pain compared to those with high income. People with higher income were more likely to have chronic rhinitis. Non-autonomy at work was associated with higher prevalence across all disease groups. Skilled and unskilled jobs were associated with higher prevalence across all disease groups.

**Table A1:** Most frequent chronic diseases in the Korea Health Panel (2010–2015).

	2010–2011				2012–2015		
No	Chronic Disease	KCD-6	Freq (%)	No	Chronic Disease	ICD-10	Freq (%)
1	Hypertension	19,031	4893 (14.3)	1	Hypertension	I10	12,141 (12.22)
2	Arthritis	23,021	2482 (7.17)	2	Hyperlipidemia	E78	5246 (5.28)
3	Gastritis	21,051	1819 (5.25)	3	Diabetes	E14	4811 (4.84)
4	Diabetes	14,021	1809 (5.22)	4	Gastritis	K29	4531 (4.56)
5	Allergic rhinitis	20,081	1290 (3.72)	5	Allergic rhinitis	J30	4518 (4.55)
6	Osteoporosis	23,091	1105 (3.19)	6	Arthritis	M14	4400 (4.43)
7	Back pain	23,072	1054 (3.04)	7	Osteoporosis	M81	3200 (3.22)
8	Hyperlipidaemia	14,081	1042 (3.01)	8	Disc disorder	M54	3178 (3.20)
9	Disc disorder	23,061	1039 (3.00)	9	Arthritis	M19	3119 (3.14)
10	Cataract disease	17,041	887 (2.25)	10	Cataract disease	H26	2876 (2.89)
11	Gingivitis	21,022	780 (2.25)	11	Gingivitis	K05	2427 (2.44)
12	Nail diseases	11,282	613 (1.77)	12	Disc disorder	M51	2148 (2.16)
13	Atopic dermatitis	22,022	530 (1.53)	13	Nail disease	B35	1880 (1.89)
14	Dry eye	17,101	528 (1.52)	14	Spondylopathesis	M48	1650 (1.66)
15	Rhinitis	20,082	517 (1.49)	15	Prostate problem	N40	1620 (1.63)
16	Prostate problem	24,081	456 (1.32)	16	Eye disease	H18	1537 (1.55)
17	Asthma	20,121	441 (1.27)	17	Atopic dermatitis	L20	1388 (1.40)
18	Allergy	22,020	425 (1.23)	18	Muscular disease	M79	1260 (1.27)
19	Dental caries	21,011	413 (1.19)	19	Dental caries	K02	1105 (1.11)
20	Disc disorder	23,074	350 (1.01)	20	Asthma	J45	1045 (1.05)
21	Angina	19,051	349 (1.01)	21	Disc disorder	M50	1039 (1.05)
				22	Sleep disorder	G47	1033 (1.04)
				23	Major depressive disorder	F32	996 (1.00)

ICD-10: the 10th revision of the International Statistical Classification of Diseases and Related Health Problems; KCD-6: the 6th revision of the Korean Classification of Diseases. Disease codes in 2010–2011 were recorded using 6-digit numbers of KCD-6, a medical classification list by the Statistics Korea, the national statistics office of Korean government.
